# Evaluation of a Study Protocol of the Application of Humor Interventions in Palliative Care Through a First Pilot Study

**DOI:** 10.1089/pmr.2023.0014

**Published:** 2023-09-11

**Authors:** Lisa Linge-Dahl, Sonja Heintz, Willibald Ruch, Maria Bley, Eckart von Hirschhausen, Lukas Radbruch

**Affiliations:** ^1^ Department of Palliative Medicine, University Hospital Bonn, Bonn, Germany.; ^2^ Faculty of health, School of Psychology, University of Plymouth, Plymouth, United Kingdom.; ^3^ Department of Psychology, University of Zurich, Zurich, Switzerland.; ^4^ Foundation “Humor Hilft Heilen” (Humor Helps to Cure), Bonn, Germany.; ^5^ Center for Palliative Care, Helios Hospital Bonn/Rhine-Sieg, Bonn, Germany

**Keywords:** end-of-life, humor, intervention, palliative care

## Abstract

**Background:**

Humor and laughter might have an alleviating effect on pain threshold and enhance coping and building relationships. However, randomized controlled studies in palliative care have struggled with high percentages of attrition and missing values.

**Objectives:**

We aimed to evaluate a study protocol through a pilot study for the evaluation of a multistage humor intervention with psychological and physiological outcome parameters that may be applied successfully in a palliative care environment.

**Design:**

This pilot study utilized a pre–post design. The inclusion of a control group for the final study setting recruiting 120 patients is planned.

**Setting/Subjects:**

The study was a monocenter study in a clinic for palliative care in Germany. All patients were eligible for recruitment. Seven patients were recruited for the pilot study.

**Measurements:**

Interventions were developed using a humor training for psychiatric patients. Quantitative sensory testing for pain threshold testing and questionnaires on humor as a character trait, pain intensity, life satisfaction, and symptom burden were planned to be evaluated before and after three humor interventions.

**Results:**

The feasibility of the original study design was re-evaluated after pilot testing. Only two out of the seven patients were able to complete two interventions, requiring modification. Fewer questionnaires, less complex physiological testing, and reduction from three to two interventions were then planned.

**Conclusion:**

The initial planned research methodology must be adjusted for patients with high symptom burden. In the experimental group of the final study setting, the effects of one to two interventions will be evaluated measuring oxytocin levels in saliva and using standardized questionnaires to determine cheerfulness, life satisfaction and symptom burden, as well as assessing as-needed medication.

**Trial registration:**

DRKS00028978 German Registry of Clinical Studies.

## Background

Defining humor presents a challenge due to its multifaceted nature with a wide range of perspectives and applications. Humor can be self-generated, appreciated, employed as a coping mechanism, convey aggressive content, be practiced as a cheerful and composed attitude toward life, and can be both a component of one's character and a situation-specific state. A definition that comes closest to what we aimed to foster in this study is the one by Ruch,^1^ “Humor is associated with a personality-based cognitive-emotional style of processing situations and life in general, characterized by the ability to find positive aspects even in negative situations (dangers, self-threats, etc.), remaining calm and composed, and even being able to smile or react with amusement, at least to some extent.”

Humor and health might be related.^2^ Scientific proof for this link is growing, and there are some indications of a beneficial effect of laughter and humor interventions for adult patients.^3^ A meta-analysis of randomized controlled trials of laughter and humor interventions described a significant decrease of depression, anxiety, and sleep quality in adults.^4^ Pinna et al.^5^ and Linge-Dahl et al.^6^ have summarized the limited studies exploring humor, health, and palliative care. They suggest that palliative care professionals are frequently using humor. Results from this review suggesting patients' coping,^7–9^ relationship-building,^10^ and psychotropic dose burden^11,12^ may benefit. This helps the patients by gaining a different perspective of their own dying process.^13,14^ However, the systematic reviews described that standardized evaluations, including a control group, has only been implemented in one of the studies.^15^ Results are also limited as humor interventions during the last days of patients' lives are ethically problematic.

Research has shown that humor interventions may be designed in various ways.^16–19^ Humorous videos,^20,21^ clown visits,^22–24^ laughter yoga,^25,26^ and other personalized interventions^27^ have all shown benefit to some degree. Group and individual interventions^28,29^ and the use of different kinds of humor^30–33^ have been tested. Staff in palliative care institutions show a strong gatekeeper barrier toward new or potentially burdening experiences for their patients.^34^ Palliative care professionals' use of humor and laughter within teams has also been documented^35^ as strongly developed.

The reproducibility of humor interventions is challenging due to the subjective and context-dependent nature of humor. The perception and response to humor can vary significantly among individuals and cultural backgrounds, making it difficult to establish standardized protocols and consistent outcomes across studies. This issue has been acknowledged in the field,^4,36,37^ emphasizing the need for rigorous methodology and replication studies to enhance the reliability and generalizability of results in humor intervention research.

There are significant barriers to performing clinical research in palliative care, especially with randomized controlled studies.^36,38–40^ High levels of attrition have been reported in various patient groups receiving palliative care services such as with advanced cancer,^41^ heart failure,^42^ and chronic obstructive pulmonary disease (COPD).^43^ Patients did not want to take part in studies with “too much record keeping” and reported being “too tired or to sick” (p.77; Ref.^36^). Chen et al.^44^ asked researchers from the field about their experiences and found that limited funding and work capacities, the challenging nature of the field, and discomfort in relation to the topic also create barriers. Preston et al.^45^ suggested attrition in palliative care clinical trials should be expected. Missing values and attrition in the results should be carefully analyzed.

Some outcome parameters that are often used in palliative care research are quality of life, pain, and overall symptom burden.^46–49^ Positive psychology research rather focuses on outcomes such as life satisfaction and personality traits: for example, cheerfulness, playfulness, or preferred humor styles.^30,50^ Oxytocin might be used as an indicator of well-being.^51^ Radioimmunoassay (RIA) oxytocin has previously been described by de Jong et al.^52^ as a potential analysis method.

## Methods

### Aim of the study

This pilot study aimed to explore a methodology to evaluate the psychological and physical effects of humor interventions on patients treated in a palliative care unit. We selected evaluation instruments that minimized patient burden and attrition. Enhanced cheerfulness is potentially influenced by humor interventions and was, therefore, included.^53^

### Trial design and study setting

A pilot testing was performed to prepare a monocenter randomized controlled clinical trial. Assessment involved testing the effect of three humor interventions on patients. The evaluation encompassed life satisfaction, character strengths, cheerfulness, burden of symptoms, stress, pain sensation, order of as-needed medication, and pain threshold. The control group would receive standard palliative care.

### Recruitment and randomization

Participants were recruited from the palliative care ward of the University Hospital Bonn. Participants had to be conscious, orientated, adequately alert to respond to the questionnaires, and had to speak German fluently. The inclusion criteria fulfillment for each patient was discussed with the ward staff. Potential participants were randomized to intervention group or control group using a simple randomization list constructed with the random number generation function in Microsoft Excel. The study was not blinded. To test for a medium effect with α > 0.5 and a power of 0.7 (Cohens *d*), 240 patients would be required: 120 each in the intervention and control groups. All participants had to provide written informed consent. If inclusion criteria were not met patients that had not completed the assessments could still receive a humor intervention as compassionate use, according to the mission of Humor Hilft Heilen (Humor helps to cure) is to provide humor interventions to anyone who wants to receive it.

Control group data collection was scheduled on alternate days, to avoid inadvertent contact with the humor interventions in progress.

### Intervention visits and evaluation instruments

Data collection included measurements of character strengths, cheerfulness, symptom burden and well-being, life satisfaction, pain sensation, and pain threshold.

**FIG. 1.**
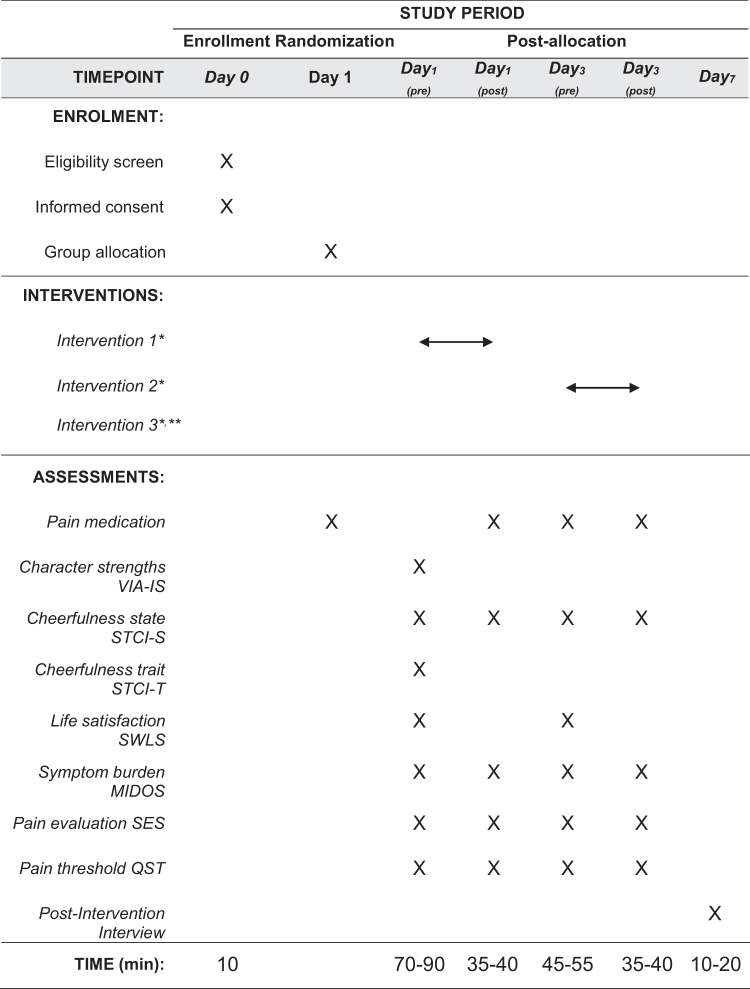
SPIRIT flowchart of pilot test sequence intervention group. *Each intervention took 20–30 minutes. **Same data collection scheme as intervention 2 on day five.

The humor intervention was based on the Humor Habits program from McGhee,^54^ which has been adapted by Falkenberg et al.^55^ for patients being treated in an inpatient psychiatric setting. It was planned to take place in three separate individualized sessions. Two trained humor coaches by the foundation Humor Helps to Cure (Humor Hilft Heilen) implemented the intervention. If possible the intervention was repeated on days three and five (or one week after the first intervention according to the availability of the humor coaches, see Fig. 1) following a multistage model.^55^ Each intervention was scheduled to take ∼30 minutes. The first intervention included the following elements: remembrance of a funny episode during childhood to find the patient's preferred humor style and then providing humor according to that style for the participant.

The second and third intervention focused on finding humorous aspects in the current situation, producing humor and applying humor in everyday life. Given that the processing speed of the elements per person could vary significantly, the allocation into first, second, and third intervention was tailored to the individual pace of the patients. The coaches used various requisites (such as musical instruments, pencils, and a folding rule) but mostly they communicated and used imagination to create humorous interactions. Both coaches were educated as hospital clowns and play at least one instrument; one studied at a circus school in Brussels, Belgium and is a trained actress, the other studied at the clown school Hannover and is a certified social worker.

After entering, the humor coaches always explored the mood of the patient first and then tried to find a matching tone to communicate. They asked every patient a couple of questions regarding the biography and a humorous anecdote from the patients' childhood to get to know the patient's preferred humor style. Subsequently they tried to find humorous aspects in the current situation using everything available in the room or finding something funny in the information the patient had given. If the patients were still at the palliative care ward the coaches prepared a second and potentially third visit based on the first visit. If it had not happened already, they encouraged the patient with tailored motivations to engage and produce humor themselves.

Unstructured field notes with time stamps were taken to document the interaction with and the reactions of the patients. Qualitative data analysis using MAXQDA software was planned for the field notes. Immediately after the intervention questionnaire assessment was repeated.

Cheerfulness was measured using the state-trait-cheerfulness-inventory—trait part and state part (STCI-T and STCI-S).^56–59^ The STCI-T (30 items) and STCI-S (18 Items) consist of cheerfulness, seriousness, and bad mood scales, which are built from sum scores of 10 (STCI-T) and 6 (STCI-S) items, respectively. The investigator aided questionnaire completion by reading questions to patients or supervising the patients' reading and responses, depending on patient performance level. Symptom burden and well-being were assessed using the Minimal Documentation System for patients in palliative care (MIDOS).^60^ MIDOS uses categorical scales, with 10 items on physical and psychological symptoms and one item on general well-being. Life satisfaction was measured using the Satisfaction with Life Scale (SWLS)^61^ it comprises five items whose sum score indicates current life satisfaction. All questionnaires are listed in the Supplementary Material.

Assessment of humor as character trait using the Values in Action Inventory of Strengths (VIA-IS)^62^ with 240 items and perception of pain using the Schmerz-Evaluations-Skala (pain evaluation scale) (SES)^63^ consisting of 24 items were included.

Measurement of the pain threshold using an extract of the quantitative sensory testing (QST) system.^64^ QST is a standardized method for testing perception- and pain-thresholds using different mechanical stimuli and by that, the functioning of the somatosensory system can be characterized. QST puts calibrated stimuli on the skin and underlying tissue to test the perception and pain-threshold or pain-tolerance-threshold using nonpainful and painful stimuli.^46,64–67^ For this study, three out of the seven standardized tests were included. The mechanical detection thresholds (von Frey filaments and a 64-Hz tuning fork), mechanical pain sensitivity (Pinprick stimuli, brush, Q-Tip, cotton wool) and the pressure pain threshold, to reduce the burden on the participants. It was estimated that the three QST tests would take a maximum of 30 minutes. All tests and questionnaires added up to 328 items and a total duration of preintervention testing of more than one hour. The post-interventional status would take ∼30 minutes.

Information on as-needed medication administered before and after the interventions was extracted from the patients' medical records. This information aimed to determine whether observed differences in symptom intensity were related to medications.

The same test batteries were repeated before (STCI-S, SWLS, MIDOS, SES, and QST) and after the second and third intervention (Fig. 1). A semistructured interview was planned two days after the third intervention to explore the patient's experience and perceived intervention burden and benefit. The interview guideline was divided into three main categories with seven open-answer questions. Answers were documented on paper by the researcher who conducted the interview and the interventions. We planned to use MAXQDA for qualitative data analysis.

### Ethics

This study was approved by the ethics committee of the University Hospital Bonn (No. 003/16). Every participant will be asked to give written informed consent before being included in the study. The informed consent document and committee approval letters are obtained.

## Results of Pilot Study

Seven patients were recruited for a pilot study, but only three were able to complete the pain threshold measurement. Two agreed to complete the related questionnaires and take part in two interventions; one completed all the test instruments before and after the two interventions. This patient also agreed to the assessment of the pain threshold (QST) and questionnaires after the second intervention. The other patients did not consent to repeat QST or did not complete questionnaires. One patient agreed to the day seven interview.

All patients commented on the questionnaires as being too long, especially the SES to having a number of redundant questions and being difficult to understand after about half of the items. The participating researcher observed reduced levels of concentration and alertness toward the end of data collection and a negative mood swing after the completion of the SES. The application of QST was commented as very uncomfortable by the three patients who agreed to take part in the procedure. Patients complained that they had to fill out the same questionnaires before and after the intervention in all cases.

## Discussion

### Limitations

The interventions were standardized to a limited extent and otherwise individualized for each patient, resulting in restricted methodological transferability and a low generalizability of the findings.

As previously outlined in the background section, humor encompasses a wide range of manifestations, making its definition and measurement challenging. This aspect further impacts the transferability of results.

The first challenge with the initiation of the study was to overcome staff's gatekeeper function, members of the clinical team voiced concerns a large portion of eligible patients had cognitive impairment and advanced disease that should preclude them from participation. We instituted ∼15-minute educational dialogue sessions during staff meetings to educate the clinical teams about the pilot study. Close cooperation with the senior physician and the lead nurse was maintained in the adaption process of the study protocol after the pilot testing.

The control group would be more meaningful if they received an intervention such as reading to them or showing a video, which uses the same amount of time and attention as the humor intervention. No patients from the control group were included in the pilot test. However, for the final study, the intention is to provide the best palliative care for all patients. This could potentially introduce bias due to additional attention given to the intervention group.

The functional status of different patients may vary significantly, due to differences in underlying diseases and stages of illnesses. Use of a staging system could help to standardize the impact of the disease.

As Blum et al.^36^ previously reported, there is bias toward exclusion of patients with high symptom burden. This limitation affects the generalizability of the findings to all palliative care patients.

In addition, the transferability of results is constrained by the fact that the study was conducted in a single-center setting. Since the study was not blinded, there is also a risk of bias due to potential variations in the researchers' interactions with the intervention and control group.

Implementing interviews within the study framework was challenging due to temporal constraints. Patients who were fit enough to participate in data collection were often discharged home or transferred to a hospice within the seven-day study period.

Finally, patient expectations surrounding a humor intervention may have been a source of bias in our pilot study. One of the patients, for example, voiced a concern her physical and mental state may inhibit her sense of humor. Although this ultimately was not the case for her, such anticipation itself could affect outcomes. Therefore, future studied interventions will begin with a careful assessment of the patient's prior expectations and current situation to minimize potential bias introduction.

### Discussion of changes after pilot testing

The literature on humor interventions in palliative care has primarily been focused on workshops and interventions for staff.^34,35^ However, humor interventions may have a meaningfully supportive role for patients receiving palliative care services. This pilot study supported literature findings^36^ suggesting extensive research data collection is excessively burdensome for those facing serious illness. Higher symptom burdens and increased time obligations restrict these patients' capacity to participate in extended research-related activities. We considered the cognitive and physical limitations often experienced by this population when creating the pilot study protocol.

However, its results demonstrated more challenges than anticipated. Our pilot study supported the available literature^39,40^ suggesting our single center would be unlikely to recruit sufficient statistical power. However, research on complex interventions^68^ such as humor therapy may be difficult to evaluate in multicenter trials, as these interventions are provided by highly skilled specialists who would need to be trained in advance to maximize comparability between therapists and centers. It was determined the semistructured interview planned for two days after the third intervention (day seven) was excessively burdensome for this patient population.

We plan to involve our specialized homecare palliative care team (SAPV) in the study, as home-treated patients in our services often have more resources and are in healthier condition. This may facilitate the participation in interventions and more complete datasets. The palliative care inpatient consultation team in the hospital is working on transferring patients with palliative care needs earlier, so that we can reduce the proportion of patients in the terminal phase of dying who are being treated at the palliative care ward. This team is working toward early integration of palliative care, including earlier transfer to the palliative care unit for patients with complex problems and needs. This should lead to more patients receiving crisis intervention with subsequent transfer to other care settings and less imminently dying patients in the palliative care unit. This in turn should lead to a higher percentage of patients eligible for humor interventions.

Because of the high attrition rate in the pilot testing some instruments were removed from study setting. This study found hints that completing the SES^63^ increased patients' negative mood. Therefore, when we had to decide on shortening data collection to reduce attrition, the complete scale was removed from the study setting. The QST^64^ caused a significant physical burden and the testing elicited pain sensations in patients, who already suffered from disease-related pain to some extent. Therefore, we decided to exclude QST from the final study, as we deemed the additional burden as ethically inappropriate.

The VIA-IS, with its 240 items, was too long for patients in our palliative care unit to complete. Even though having a comprehensive profile on the character strengths of all participants would have provided valuable information, implementation was not feasible due to resource and ethical considerations. The interview was also hardly conducted due to discharge, illness progression, fatigue, or other reasons. These modifications reduced the preintervention assessment from ∼60 to 30 minutes and the post-intervention from 40 to 10 minutes.

**FIG. 2.**
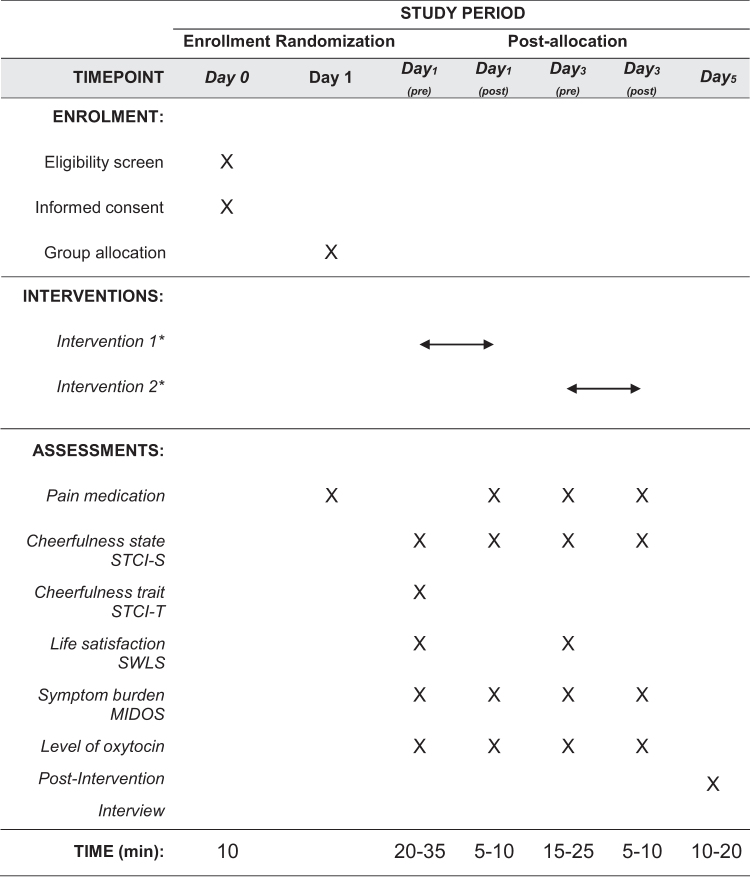
SPIRIT flowchart of final test sequence intervention group. *Each intervention took 20–30 minutes.

The questionnaires that remained in the study protocol after pilot testing were STCI-T, STCI-S, SWLS, and MIDOS (Fig. 2) because the number of items of these instruments seemed manageable for the patients. We included the STCI-T in the study because it has significantly fewer items compared with the VIA-IS and allows for checking statistical equality between the intervention and control groups. The STCI-S, as the main variable for potential mood changes, had to be retained.

We included life satisfaction, measured by the SWLS, because it has been widely used in previous studies, consists of only five items, and enables us to compare our results with others' research. We kept the MIDOS for evaluating the burden of symptoms since patients found it less burdensome than the SES during pilot testing. Including this medical evaluation instrument in the test battery was valuable for our research concept. Finally, assessment of the effect of one to two humor interventions on 120 patients, evaluating life satisfaction, cheerfulness, burden of symptoms, stress, order of as-needed medication and oxytocin levels in saliva was planned.

### Potential alternative physiological parameter

Oxytocin has been suggested as a potential indicator of well-being, as it is involved in social bonding, positive emotions, and stress regulation.^69,70^ Research has shown that higher levels of oxytocin are associated with enhanced social interactions and improved mental health outcomes.^71,72^ However, it is important to note that the relationship between oxytocin and well-being is complex, and further studies are needed to fully confirm its role as a valid indicator of well-being. The laboratory regulations prohibit saliva collection for oxytocin measurements if patients have multiresistant infections.

After completing the questionnaire, a study nurse would collect saliva by having the patient chew on a cotton wool roll for at least 60 seconds. The sample would then immediately be placed on dry ice at −80°C and then stored in a refrigerator at −80°C. Samples would then be shipped to the laboratory by courier service every six months. The salivary oxytocin level could be analyzed before and immediately after the humor interventions. For each sample 300 μL of saliva would be evaporated (Concentrator, Eppendorf, Germany), and 50 μL of assay buffer would be added, followed by 50 μL anti-oxytocin rabbit antibodies.

The detection limit of the RIA is 0.1–0.5 pg/sample; the intra- and interassay variabilities were <10%. Plasma samples (0.5 mL) were kept at −20°C until extraction using heat-activated LiChroprep^®^ Si60 (Merck) at 690°C for three hours. Twenty milligrams of LiChroprep Si60 in 1 mL distilled water are added to the sample, mixed for 30 minutes, washed twice with distilled water and 0.01 mol/L HCl, and eluded with 60% acetone. The evaporated extracts and evaporated saliva samples (0.3 mL) are analyzed for oxytocin together in a highly sensitive and specific RIA.

## Conclusion

Our pilot study revealed some unanticipated barriers for participation and potential biases that could be minimized further. We were able to utilize these results to more efficiently develop a protocol for a vigorous study that will enhance participation and optimize outcome reliability. Patients receiving treatment in the palliative care unit have a limited remaining life span, thus slimming down the humor intervention with the reduction from three to two interventions and condensing the content represents one of the most crucial improvements resulting from the pilot testing.

## Supplementary Material

Supplemental data

## Supplementary Material

Questionnaires

## Abbreviations Used

COPD: chronic obstructive pulmonary disease
MIDOS: minimal documentation system for patients in palliative care
QST: quantitative sensory testing
RIA: radioimmunoassay
SAPV: specialized homecare palliative care team
SES: Schmerz-Evaluations-Skala (pain evaluation scale)
STCI-S: state-trait-cheerfulness-inventory—state part
STCI-T: state-trait-cheerfulness-inventory—trait part
SWLS: satisfaction with life scale
VIA-IS: values in action inventory of strengths
